# Two New Species and Two New Records of the Lichen-Forming Fungal Genus *Peltula* (Ascomycota: Peltulaceae) from China

**DOI:** 10.3390/biology11101518

**Published:** 2022-10-17

**Authors:** Qiuxia Yang, Jason Hollinger, Steven D. Leavitt, Xinli Wei

**Affiliations:** 1 State Key Laboratory of Mycology, Institute of Microbiology, Chinese Academy of Sciences, Beijing 100101, China; 2 Herbarium, Western Carolina University, Cullowhee, NC 28723, USA; 3 Department of Biology, Brigham Young University, Provo Main Campus, Provo, UT 84602, USA

**Keywords:** biodiversity, biological soil crusts, lichens, Lichinales, molecular phylogeny, *Peltula*, semi-arid, semi-humid, taxonomy

## Abstract

**Simple Summary:**

This paper reports on two new species and two new records of the lichen-forming fungal genus Peltula Nyl., highlighting progress in our investigation of Chinese Peltula spp. The external morphology, anatomy, and molecular systematics were comprehensively analyzed to support the species delimitation. The results contribute to the knowledge of species diversity and geographical distributions of Peltula species in China.

**Abstract:**

In this study, two new species, *Peltula confusa* Q.X. Yang & X.L. Wei, sp. nov., growing in a dry microenvironment within a semi-humid area, and *Peltula subpatellata* Q.X. Yang & X.L. Wei, sp. nov., occurring in arid and semi-arid regions, are described. In addition, two species, *P. polyspora* (Tuck.) Wetmore and *P. obscuratula* (Nyl.) Poelt ex Egea, are recorded for the first time in China. All four species are described based on morphological, anatomical and molecular data. *Peltula confusa* is characterized by a lighter, brighter, and more yellowish upper surface than other species of this genus, with discs concentrated in the central part of squamules, and a thick lower cortex. *Peltula subpatellata* can be distinguished from *P. patellata* (Bagl.) Swinscow & Krog by its non-thickened and sometimes darkened margins and sometimes the presence of peltate squamules. Phylogenetic analysis based on DNA sequences of four loci (ITS, nrSSU, nrLSU, and RPB2) demonstrates the placement of these species within *Peltula*, and supports current species delimitations. We suggest that the growth substrate should be considered as an additional characteristic for species delimitation.

## 1. Introduction

The lichen genus *Peltula* (Lichinales, Lichinomycetes, Ascomycota) has grown to comprise more than 60 species since the type species, *Peltula radicata* Nyl., was described by Nylander in 1853. After being established, this genus name was not used further for a long period of time and many species that are now accommodated in the genus *Peltula* were described as part of the genus *Heppia* Naeg. It was not until 1935 that *Peltula* was mentioned again and began to be used as a separate genus [1]. Based on the development of ascocarps, the unique structure and function of the ascus, and numerous ascospores in the ascus, Büdel considered *Peltula* to be a monophyletic genus and established a new family, Peltulaceae, for it, including four growth types: leaf-like, squamulose, crustose and fruticose [2]. Büdel and Kalb added the genera *Neoheppia* Zahlbr. and *Phyllopeltula* Kalb to the family, respectively [3,4]. Kauff et al. confirmed that Peltulaceae was a monophyletic family using phylogenetic analysis, and combined the two above-mentioned genera, i.e., *Neoheppia* and *Phyllopeltula*, into *Peltula* [5]. Based on the newest revision of Peltulaceae, *Peltula* is characterized by five growth forms of the thallus, viz. peltate, squamulose, subfruticose, subfoliose, and crustose. 

About 20 species of *Peltula* from China have been reported. Previously, *P. euploca* (Ach.) Poelt, *P. impressula* (H. Magn.) N.S. Golubk, *P. minuta* (H. Magn.) N.S. Golubk, *P. placodizans* (Zahlbr.) Wetmore and *P. tortuosa* (Nees) Wetmore were known to be present in arid and semi-arid desert regions [6,7,8]; *P. applanata* (Zahlbr.) J.C. Wei was described as a new species from Guizhou province, which is a humid region [9]; *P. bolanderi* (Tuck.) Wetmore, *P. clavata* (Kremp.) Wetmore, *P. coriacea* Büdel, Henssen & Wessels, *P. corticola* Büdel & R. Sant., *P. euploca*, and *P. placodizans* were identified in Hong Kong and Taiwan [10,11,12]; and *P. cylindrica* Wetmore, *P. euploca*, *P. lobulate* Q.X. Yang & X.L. Wei, *P. polycarpa* Q.X. Yang & X.L. Wei, *P. polyphylla* Q.X. Yang & X.L. Wei, *P. pseudoboletiformis* Q.X. Yang & X.L. Wei, and *P. submarginata* Q.X. Yang & X.L. Wei were reported in humid and semi-humid areas [13,14]. Such a high diversity of species indicates that China is an important reservoir of *Peltula*, and it is likely that more species may be found.

We conducted an investigation into lichens in several provinces across China and collected numerous specimens, among which *Peltula* species were identified. The *Peltula* specimens collected from Beijing, Inner Mongolia, Gansu, and Ningxia are mainly squamulose in growth type. Among these localities, Beijing is rich in natural forest resources and has distinct wet and dry seasons, while the other three regions are (semi) arid regions. 

## 2. Materials and Methods

### 2.1. Taxon Sampling

Specimens for this study were collected from Beijing, Gansu, Inner Mongolia, and Ningxia. The voucher specimens were deposited in the Fungarium of the Institute of Microbiology, Chinese Academy of Sciences (HMAS-L). Leica M125 (Leica Microsystems, Wetzlar, Germany) and DFC450 (Leica Microsystems, Wetzlar, Germany) dissecting microscopes were used for the morphological studies. Free-hand sections were used to study the internal morphology of the lichen thallus and ascomata. A Zeiss Axioscope2 compound microscope (Zeiss Corporation, Göttingen, Germany) with a Zeiss Axio Imager A2 (Zeiss Corporation, Göttingen, Germany) was used for the anatomical studies, and an AxioCam MRc5 camera (Zeiss Corporation, Göttingen, Germany) was used to take photographs. Spot tests were performed using K (10% aqueous solution of potassium hydroxide) and IKI (1% aqueous iodine solution with 10% aqueous potassium hydroxide, Sinopharm Chemical Reagent Co., Ltd, Beijing, China). 

### 2.2. DNA Extraction, Amplification, and Sequencing

Thirty-four fresh specimens were chosen for DNA extraction. The modified CTAB method was used as the extraction procedure [15]. In general, PCR amplifications and primers used followed the work by Kauff et al. [5]. The ITS amplification primers were ITS1 / ITS4, and ITS4 / ITS5 [16], and the ITS cycling parameters consisted of the initial denaturation at 94 °C for 2 min; 33 denaturation cycles at 94 °C for 30 s, annealing at 55 °C for 30 s, extension at 72 °C for 1 min 10 s; and a final extension at 72 °C for 2 min. Reactions were carried out in a volume of 25 µL containing 12.5 µL of 2 × Taq PCR MasterMix® (Beijing Jiangchen Biotechnology Co., Ltd, Beijing, China), 1 µL of each primer solution (10 µM), 9 of µL ddH_2_O and 1.5 µL of genomic DNA. The new sequences generated in this study were deposited in GenBank (www.ncbi.nlm.nih.gov, accessed on 27 August 2022) (Table 1)

### 2.3. Sequence Alignment

All new sequences and reference sequences downloaded from GenBank of four gene loci were included in the alignments. *Peccania terricola* H. Magn. and *Peccania* spp. were chosen as outgroups. Sequences were aligned separately for each locus using ClustalW Multiple Alignment in BioEdit v7.0.5 [17,18]. The program Gblocks v0.91b [19,20] was used to remove regions of alignment uncertainty (http://molevol.cmima.csic.es/castresana/Gblocks_server.html, accessed on 21 July 2022). 

### 2.4. Congruence among Loci

Highly supported clades (≥75% bootstrap) from single-locus trees were compared to assess the level of congruence among loci [21,22]. When there was no conflict using a 75% bootstrap value threshold, the data set was concatenated. In situations where a monophyletic group was supported with bootstrap values ≥ 75% at one locus and the same group of taxa was supported by ≤75% as non-monophyletic with another locus, the group was still assumed to be congruent [22]. Each locus was subjected to a randomized accelerated maximum likelihood (RAxML) analysis involving 1000 pseudoreplicates with RAxML-HPC BlackBox 8.2.6 [23] on the Cipres Science Gateway (http://www.phylo.org, accessed on 21 July 2022) and all four single-locus RAxML trees were compared. The conflicting gene sequences were removed based on the significant topological differences and the analysis was repeated until no further conflicts were detected. The four single-locus RAxML trees are shown in Supplementary Figures S1–S4.

### 2.5. Phylogeny of the Genus Peltula

The concatenated data set was subjected to phylogenetic analysis using RAxML-HPC v. 8.2.6 [23] and MrBayes v.3.2.6 [24,25] on the Cipres Science Gateway (http://www.phylo.org, accessed on 21 July 2022). The GTR + I + G model was selected in both ML and Bayesian analyses with 1000 pseudoreplicates. Two parallel Markov chain Monte Carlo (MCMC) runs were performed in MrBayes, each using 8 million generations and sampling every 1000 steps. A 50% majority-rule consensus tree was generated from the combined sampled trees of both runs after discarding the first 25% as burn-in. Tree files were visualized with FigTree v.1.4.2. 

## 3. Results

### 3.1. Phylogenetic Analysis

**Figure 1:**
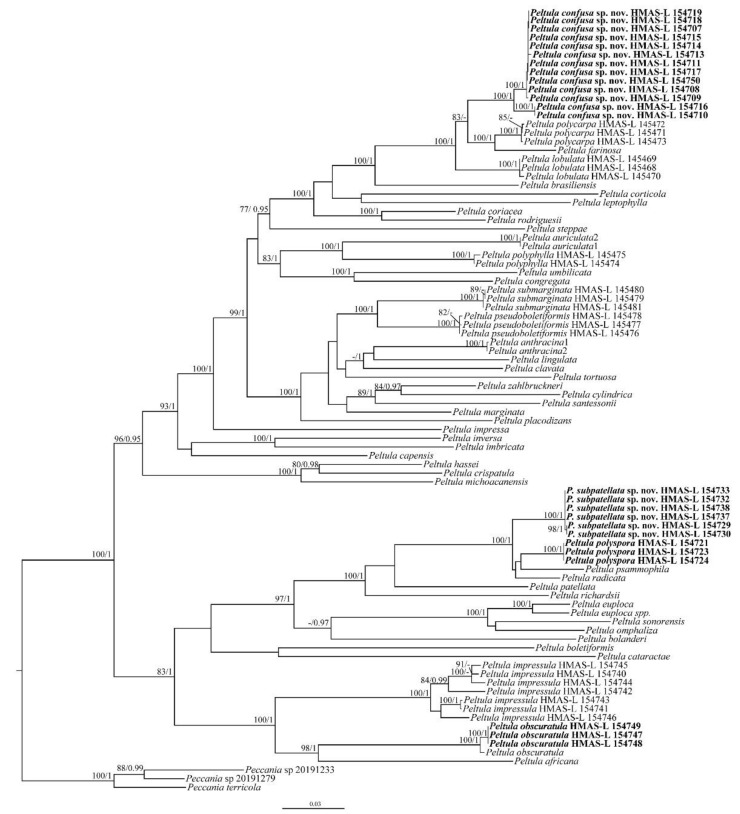
The RAxML tree of *Peltula* species based on the concatenated ITS + nrSSU + nrLSU + RPB2 data set. The numbers in each node represent bootstrap support (BS) and posterior probability (PP) values. BS values ≥ 75 and PP values ≥ 0.95 are plotted on the branches of the tree. The clades corresponding to the new species and the new records are in bold. The scale bar is 0.03 substitutions per site.

A total of 295 DNA sequences, including 104 new sequences (34 ITS, 33 nrLSU, 23 nrSSU and 14 RPB2) generated for this study, were used (Table 1). Bayesian Inference (BI) and Maximum Likelihood (ML) phylogenetic trees of the concatenated data set of the four gene markers were constructed, and they had similar topological structures. The RAxML tree is shown in Figure 1 with both bootstrap support (BS) and posterior probability (PP) values of BI analysis. The resulting tree showed two well-supported (BS = 100, PP = 1.00) branches corresponding to the two new species and almost all species had strong support. Although the new species, *Peltula confusa*, showed small genetic differences within the species, all specimens were consistent in terms of their morphology. The results also revealed the high genetic diversity of *P. impressula*. Initially, some specimens of *P. impressula* were mistakenly considered to be *P. radicata*, but all the specimens of *P. impressula* consistently had peltate squamules with small white dots (versus *P. radicata* with its fissured surface), a thin upper cortex (versus *P. radicata* without an upper cortex), and root-like rhizoids [6,8,26]. Our results also highly support the distinctness of the new species *Peltula subpatellata* from *P. patellata* and it can be easily distinguished by its morphology. The BI phylogenetic tree and four single-gene-locus RAxML trees are shown in Figures S1–S5.

### 3.2. Taxonomy

**Figure 2:**
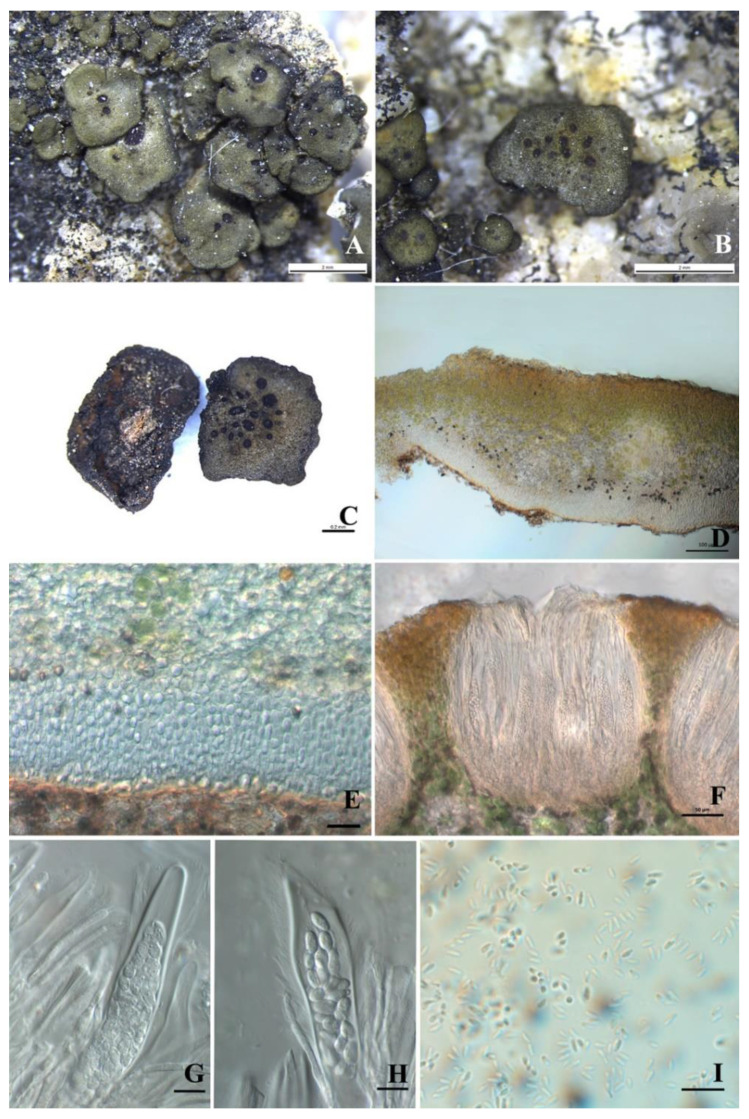
The thallus habit and anatomic structure of *Peltula confusa* (holotype). (**A**–**C**) Squamulose thallus and immersed apothecia. (**D**) Thallus section. (**E**) Thick lower cortex. (**F**) Section of apothecium. (**G**–**H**) Bitunicate ascus with a lacerate outer-wall-like gelatinous sheath. (**I**) Conidia. Bars: (**A**−**B**) = 2 mm, (**C**) = 0.2 mm, (**E**) = 20 μm, (**F**) = 50 μm, (**G**−**I**) = 10 μm.

*Peltula confusa* Q.X. Yang & X.L. Wei, sp. nov. (Figure 2).

Fungal Names: FN 571302.

MycoBank: MB845798.

Etymology: The epithet ‘confusa’ refers to the obscure characteristics of this new species.

Holotype: CHINA, Beijing, Changping District, Beijing Dayangshan National Forest Park, on the way to the summit of Xiaoyang mountain, 40°18′ N, 116°25′ E, alt 390 m, on rocks, 18 Jun 2019, Q.X. Yang & W.C. Wang 20191620 (HMAS-L 154707).

Diagnosis: The new species is characterized by a lighter, brighter and more yellowish upper surface than in other species of this genus, apothecial discs concentrated in the central part of the squamules and a thick lower cortex.

Description: Thallus squamulose, saxicolous, 2–4 mm in diameter, up to 7.5 mm, irregularly rounded, convex at first, peltate at maturity; margins down-rolled, entire to slightly lobed with a deepened color; upper surface light-yellowish green to olive green, matte and tarnished, epruinose; lower surface matte, brown, attached to the substrate by central short umbilicus; isidia and soredia absent. Thallus 250–450 μm thick, upper cortex not developed but with a yellowish epinecral layer, 5–8 μm thick; photobiont layer 100–135 μm thick, cyanobacteria unicellular, cells 7–9 μm in diameter, in clusters of 2–4 cells with a sheath; medulla composed of interwoven hyphae and round cells, 75–110 μm thick, hyphae indistinct; lower cortex paraplectenchymatous and 45–85 μm thick, made up of about 10 layers of cells, cells 5.5–12 × 3.5–5.5 μm in size. Apothecia 1–20 per squamule, concentrated in the center of squamules, discs reddish black, immersed, up to 0.45 × 0.75 mm, thalloid rim absent. Epihymenium thin or absent, yellow-brown, K-; hymenium approximately 150–175 μm tall, I+ wine red, K-, paraphyses septate, 1.8–2.7 μm wide; subhymenium approximately 70–110 μm thick; asci clavate to obclavate, bitunicate, outer wall (previously described as a lacerate gelatinous sheath) present, ascus 85–125 × 10–22μm, more than 64 spores, ascus wall, and subhymenium IKI+ blue after K pretreatment; ascospores hyaline, ellipsoid, simple, 5.5–7.9 × 2.5–3.8 μm. Pycnidia immersed, cerebriform, ellipsoid, hyaline, 1.0–1.5 × 1.6–3.4 μm.

Habitat and distribution: The species was found on sun-exposed sandstone along the road in low-altitude mountains. It was intermixed with other species of this genus and other rock-inhabiting groups. It is presently known only to occur in China.

Additional specimens examined: CHINA, Beijing, Changping District, Mangshan National Forest Park, 40°15′ N, 116°17′ E, alt 450 m, on rocks, 31 May 2019, W.C. Wang & Q.X. Yang. 20191485 (HMAS-L 154714); Beijing Dayangshan National Forest Park, on the way to the summit of Xiaoyang mountain, 40°18′ N, 116°25′ E, alt 390 m, on rocks, 18 Jun 2019, Q.X. Yang & W.C. Wang, 20191619 (HMAS-L 154710), 20191621 (HMAS-L 154711), 20191622 (HMAS-L 154708), 20191623 (HMAS-L 154709), 20191625 (HMAS-L 154712), 20191646-2 (HMAS-L 154715), 20191648 (HMAS-L 154713); Mentougou District, Qingshui Town, Bamuyan Village, Baihuagu, 39°51′12″ N, 115°33′29″ E, alt ca. 765 m, on rocks, 31 Aug 2019, X.L. Wei et al. 20191928 (HMAS-L 154716), 20191944 (HMAS-L 154717); behind Xishanhong Inn, 39°51′12″ N, 115°33′3″ E, alt 744 m, on rocks, 1 Sep 2019, X.L. Wei et al. 20192000 (HMAS-L 154718), 20192011 (HMAS-L 154719), 20192019 (HMAS-L 154720), 20192022 (HMAS-L 154750).

Notes: This species was only found in Beijing, and it is very abundant in this area. We obtained many specimens of this species, and frequently free-living cyanobacteria are found to be associated with the thallus. Considering the seasonally wet and dry climate, we assumed that the rain washed the cyanobacteria from elsewhere, a phenomenon that is also common in *Peltula pseudoboletiformis*, another species we collected. *Peltula confusa* grows with other species of this genus, i.e., *P. lobulata* and *P. placodizans*, but the upper surface of the new species is lighter, brighter, and more yellowish, so they can be easily distinguished by their thallus color. 

*Peltula confusa* often does not have prominent features, such as appendage structures or a special thallus outline. It is similar to *P. sonorensis* Büdel & T.H. Nash, which also has a simple thallus; however, the new species has a matte and tarnished upper surface, the absence of an upper cortex, a thicker lower cortex (45–85 μm, 10 layers of cells), and reddish black apothecial discs concentrated in the central part of the squamules, while *P. sonorensis* has a smooth upper surface with a primitive upper cortex, a thinner lower cortex (18–30 μm, 4–5 layers of cells), and yellow-brown discs randomly distributed within the squamules [27].

In the phylogenetic tree, *Peltula confusa* and the several rock-inhabiting squamulose species are clustered into a clade, among which *P. polycarpa* and *P. lobulata* are also distributed in the Beijing area, indicating the three species live in a similar habitat and have evolved from a common ancestor [14].

**Figure 3:**
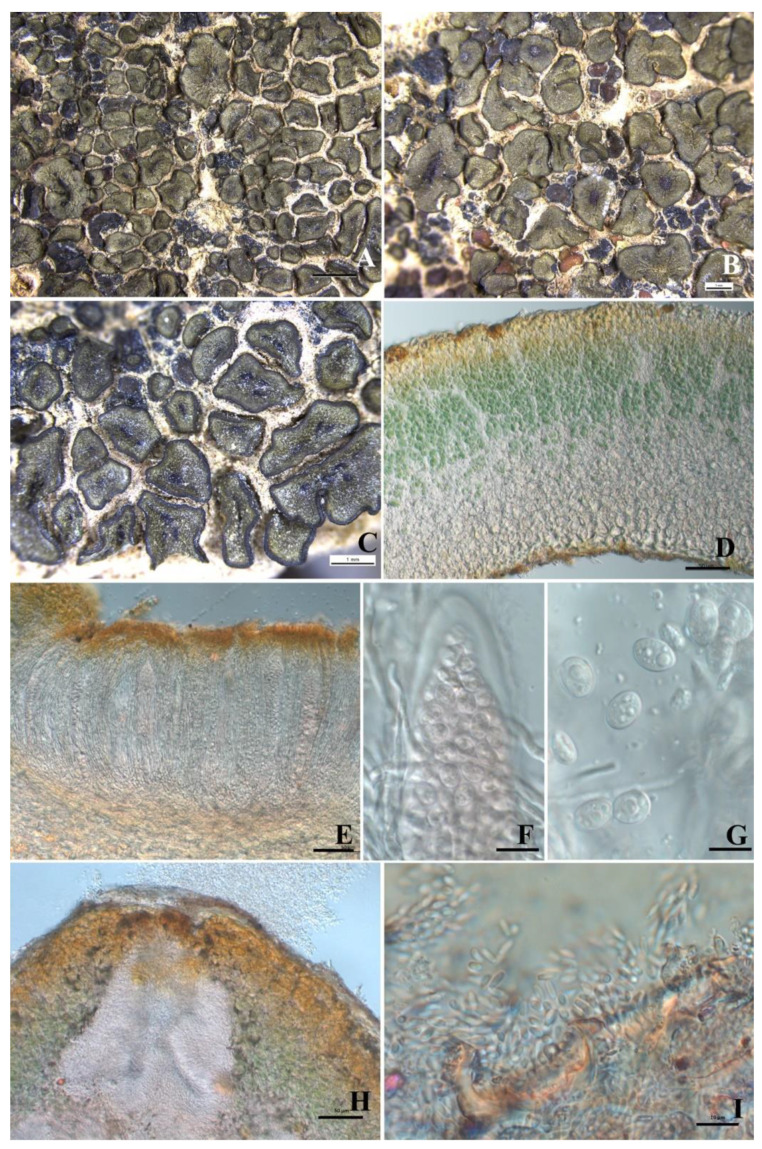
The thallus habit and the anatomic structure of *Peltula subpatellata* (holotype). (**A**–**C**) Squamulose thallus and immersed apothecia. (**D**) Thallus section. (**E**) Section of apothecium. (**F**) Biunicate ascus with a lacerate outer-wall-like gelatinous sheath. (**G**) Elliptical ascospores with oil drop. (**H**) Pycnidia. (**I**) Conidia. Bars: (**A**) = 2 mm, (**B**−**C**) = 1 mm, (**D**−**E**) = 50 μm, (**F**−**G**) = 10 μm, (**H**) = 50 μm, (**I**) = 10 μm.

*Peltula subpatellata* Q.X. Yang & X.L. Wei, sp. nov. (Figure 3).

Fungal Names: FN 571303.

MycoBank: MB845799.

Etymology: The epithet ‘subpatellata’ refers to the similarity of this new species to *Peltula patellata* in terms of its morphology.

Holotype: CHINA, Ningxia, Zhongning County, Shangliushui Village, Qingshangen, 37°4′25″ N, 105°23′32″ E, alt 1710 m, on sandy soil, 19 Jan 2019, X.L. Wei et al. 20190143 (HMAS–L 154733).

Diagnosis: This new species is different from the similar species *P. patellata* due to its non-thickened and sometimes darkened margins, and sometimes the presence of peltate squamules.

Description: Thallus squamulose, terricolous; squamules 1.5–2–(3.5) mm in diameter, orbicular to irregularly rounded, sometimes angulate, plane to deeply concave, older ones peltate and cracked; margins entire or slightly lobed, usually upturned and darker; upper surface smooth to matte, grey olive-green, usually pruinose, pruina yellow; lower surface covered with dense hyphae, attached to the substrate by a tuft of rhizoids, hyphae pale brown, septate; isidia and soredia absent. Thallus 200–407 μm thick; upper cortex not developed but with a yellowish epinecral layer, 15–25 μm thick; photobiont layer 70–150 μm thick, cyanobacteria unicellular, cells in clusters of 1–4 cells; medulla thin and unclear, 30–80 μm thick, composed of densely interwoven hyphae and big cells; lower cortex indistinctly delimited from the medulla, paraplectenchymatous, 70–87 μm thick, made up of 3–6 layers of big circular cells, cells up to 19 μm in diameter. Apothecia usually one per squamule, up to five, immersed in the center of concave squamules; discs open, red to reddish black, up to 0.4 × 1 mm in diameter, with prominent thalloid rim at maturity, margin concolorous with the thallus. Epihymenium yellowish brown, K+ red-violet; hymenium 140–185 μm tall, I+ wine red, K−; paraphyses septate, apices sometimes swollen, 2 μm wide; subhymenium about 30–57 μm thick; asci clavate to obclavate, 96–174 × 13–25.7 μm, bitunicate, outer wall lacerate, like gelatinous sheath, more than 100-spored; asci wall and subhymenium IKI+ blue after K pretreatment; ascospores hyaline, ellipsoid, simple, with oil drops, (6.6–)9.0–10.9 × (5–)6.7–7.9 μm. Pycnidia immersed, cerebriform; conidia ellipsoid, hyaline, 3.9–4.6 × 1.6–1.8 μm. 

Habitat and distribution: The new species was found on sandy soil in semi-arid and arid regions. It grows on stable or semi-stable soil with other soil-inhabiting species, but not on flowing sand. It is presently known only to occur in China.

Additional specimens examined: CHINA, Ningxia, Zhongning County, Shangliushui Village, Qingshangen, 37°4′25″ N, 105°23′32″ E, alt 1710 m, on sandy soil, 19 Jan 2019, X.L. Wei et al. 20190112 (HMAS-L 154732); Gansu, Baiyin City, Jingtai Town, Cuiliugou, 37°24′51″ N, 104°35′4″ E, alt 1640 m, on sandy soil, 18 Jan 2019, X.L. Wei et al. 20190016 (HMAS-L 154728); 20 Jan 2019, X.L. Wei et al. 20190050 (HMAS-L 154729), 20190053 (HMAS-L 154730), 20190063 (HMAS-L 154731); 37°25′6″ N, 104°34′56″ E, alt 1591 m, on sandy soil, 21 Oct 2020, Q.X. Yang et al. 20201261 (HMAS-L 154734), 20201262 (HMAS-L 154735); Inner Mongolia, Alxa Left Banner, Barrenberli town, on the edge of a provincial highway 218, 38°35′12″ N, 105°38′21″ E, alt 1338 m, on sandy soil, 11 Jul 2017, D.L. Liu & R.D. Liu. XL2017088 (HMAS-L 154739); Alxa Right Banner, on the mountains in the Northwest, 39°28′24″ N, 101°4′3″ E, alt 1564 m, on sandy soil, 22 Jul 2017, D.L. Liu & R.D. Liu. XL2017268 (HMAS-L 154738), XL2017279 (HMAS-L 154737); 39°32′31″ N, 101°6′34″ E, alt 1478 m, on sandy soil, 5 Jun 2018, D.L. Liu et al. ALS2018037 (HMAS-L 154736).

Notes: *Peltula subpatellata* strongly resembles the common and easily recognizable species *P. patellata* due to its flat to deeply concave squamules with ascending and darker margins and the existence of a tuft of hyphae connecting it to the substrate. The squamule margins of *Peltula subpatellata* are smooth, shiny, and not thickened, and are sometimes not obvious or darker in very mature peltate squamules; however, the squamule margins of *P. patellata* are thickened, resulting in a typical patellate form. The molecular data support *Peltula subpatellata* as a separate species, although it is closely related to *P. patellata*. Based on the four-locus phylogenetic tree, *Peltula subpatellata*, *P. polyspora*, *P. psammophila* (Nyl.) Egea, *P. radicata*, *P. patellata*, and *P. richardsii* (Herre) Wetmore form a highly supported clade, and they are all soil-inhabiting species found in (semi) arid regions. Although Kauff et al. attempted to group the genus by different types of growth forms, it was finally found that this grouping was unsuitable [5]. Our finding that species sharing the same substrates are phylogenetically related provides a new taxonomic characteristic for the group of this genus.

It should be noted that the ascospores of this new species contain oil drops. Among all the reported species in this genus, this characteristic has only been mentioned for *Peltula impressula* (reported as *P. oleifera* (H. Magn.) J.C. Wei) [6,28,29]. Based on our own observations, oil droplets are found in both the hymenium and ascospores of *Peltula impressula*. As for *Peltula patellata*, we did not find any information about oil droplets in past reports [30,31,32], but we recently noted their presence in the ascospores of a voucher specimen from the Santa Monica Mountains in Southern California, U.S.A. (*J. Hollinger* 9078, herb. Noell & Hollinger).

In the color reaction, the epihymenium of *Peltula subpatellata* turns red-violet in K. This reaction can also be seen in *Peltula clavata*, *P. obscurans* var. *obscurans* (Nyl.) Gyeln., *P. obscuratula*, *P. omphaliza* (Nyl. ex Eckfeldt) Wetmore, *P. patellata* (Upreti & Budel mentioned as *P. patellata* K− [32]), *P. polyspora*, and *P. richardsii* [29,33].

### 3.3. New Records for China

**Figure 4:**
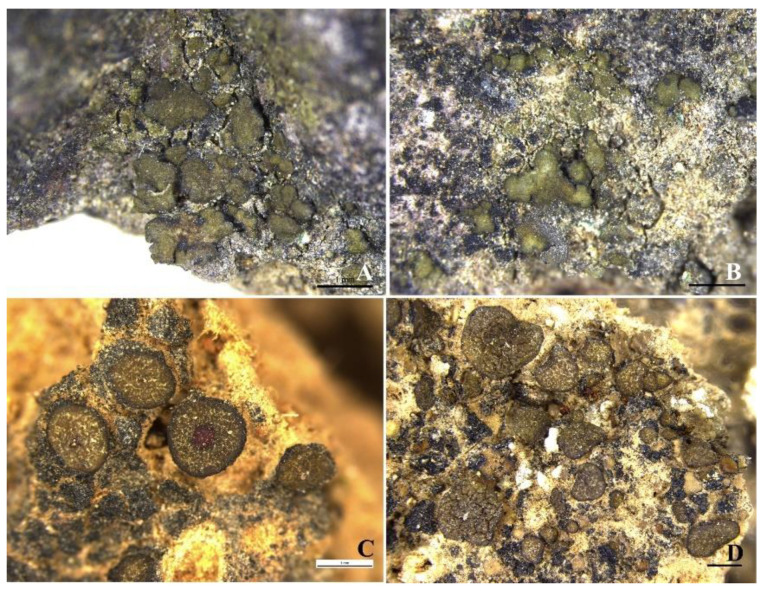
The thallus habit of *Peltula obscuratula* and *P. polyspora*. (**A**,**B**) Squamulose thallus of *P. obscuratula* (HMAS-L 154747, 154748). (**C**,**D**) Squamulose thallus of *P. polyspora* (HMAS-L 154723). Bars: (**A**−**D**) = 1 mm.

*Peltula obscuratula* (Nyl.) Poelt ex Egea Biblthca Lichenol. 31: 84 (1989). (Figure 4A−B).

Basionym: *Heppia obscuratula* Nyl., Flora, Regensburg 61: 339 (1878). Type: Not seen.

Specimens examined: CHINA, Beijing, Changping District, Mangshan National Forest Park, 40°15′ N, 116°17′ E, alt 450 m, on rocks, 31 May 2019, W.C. Wang & Q.X. Yang. 20191465 (HMAS-L 154747), 20191491 (HMAS-L 154748), 20191493 (HMAS-L 154749).

Thallus squamulose, saxicolous; squamules irregularly rounded, ca. 1 mm in diameter, convex to flat; margins undulate and lobed; upper surface dark olive-green, epruinose; lower surface matte, almost black, tightly appressed to the substrate, attached by central short umbilicus; isidia and soredia absent. Apothecia and pycnidia not seen.

*Peltula obscuratula* is reported here for the first time for China. This species is similar to *P. obscurans* in terms of its small, lobulate squamules, small umbilicus, and K+ red-to-purple epihymenium, but it differs due to its smaller ascospores [26,34].

*Peltula polyspora* (Tuck.) Wetmore, Ann. Mo. bot. Gdn 57(2): 198 (1971) [1970]. (Figure 4C−D).

Basionym: *Heppia polyspora* Tuck., Syn. N. Amer. Lich. (Boston) 1: 115 (1882). Type: Not seen.

Specimens examined: CHINA, Gansu, Baiyin City, Jingtai Town, Cuiliugou, 37°24′51″ N, 104°35′4″ E, alt 1640 m, on sandy soil, 18 Jan 2019, X.L. Wei et al. 20190012 (HMAS-L 154721); 37°23′40″ N, 104°35′54″ E, alt 1610 m, on sandy soil, 22 Apr 2019, X.M. Cheng & Q.X. Yang 20191252 (HMAS-L 154724); Ningxia, Zhongning County, Shangliushui Village, Qingshangen, 37°4′16″ N, 105°23′13″ E, alt 1738 m, on sandy soil, 19 Jan 2019, X.L. Wei et al. 20190162 (HMAS–L 154722), 20190196 (HMAS–L 154723).

Thallus squamulose, terricolous; squamules round to sometimes slightly lobed, up to 2.5 mm, flat; margins usually darker; upper surface tan-olive, matte and sometimes cracked; attached to the substrate by a tuft of hyphae; isidia and soredia absent. Apothecia one to numerous per squamule, immersed; disc reddish black; epihymenium yellowish brown, K+ red-violet; hymenium 95–180 μm; asci 90–122 × 20–31 μm, bitunicate, an outer-wall-like gelatinous sheath present; ascospores globose, 4–6 μm. Pycnidia not seen.

*Peltula polyspora* is new to China. It has previously been suggested as synonymous with *P. patellata* [31]; however, based on our molecular systematics, *P. polyspora* and *P. patellata* are distinctly different. Therefore, our results support *P. polyspora* as a separate species. In Figure 1, it can be seen that *P. polyspora* clusters closer to *P. subpatellata* than to *P. patellata.* Tuckerman mentioned that *Peltula polyspora* has numerous apothecia [35]; then, Wetmore widened the boundaries to 1–20 apothecia per squamule of this species [33]. Most of our specimens have one apothecium per squamule, which is consistent with Wetmore’s species delimitation [33]. This species is common and widely distributed in semi-arid and arid regions of China and has flatter and rounder squamules, but there was also a special case, reported from the Judean Desert, where the squamules had no definite form [29].

## 4. Discussion

In the four-locus phylogenetic analysis, the distance and genetic relationships among species of *Peltula* are clearly revealed. In particular, in species with unclear morphological boundaries, DNA sequences could resolve their phylogenetic relationships. The specimens of *Peltula subpatellata* were preliminarily identified as *P. patellata*, but the phylogenetic analysis and further morphological comparison with Jason Hollinger’s specimen (J. Hollinger 9078) revealed that these specimens represent a new species, different from *P. patellata*. Previously, there have been several reports of *P. patellata*, but in the absence of DNA sequence analysis, identification of these specimens was mainly based on their morphology alone. For example, Upreti & Budel described *P. patellata* from India as having a K- epithecium and noted that this species was similar to *P. obscurans* [32]. In fact, *P. patellata* has a K+ epithecium and it is not similar to *P. obscurans*. To reach a more natural taxonomy and species delimitation it is essential that molecular data supplement morphological observations.

## 5. Conclusions

In this study, we described two new species, *Peltula confusa* Q.X. Yang & X.L. Wei, sp. nov. and *P. subpatellata* Q.X. Yang & X.L. Wei, sp. nov., from Beijing and the northwest regions of China. These new species are rock-inhabiting in dry microenvironments within semi-humid regions, and soil-inhabiting in arid and semi-arid regions, respectively. In general, there is a trend in the substrate choice of *Peltula* spp. corresponding to the climate. Soil-inhabiting species, e.g., *Peltula impressula*, *P. radicata*, *P. richardsii*, and *P. polyspora* are distributed in (semi) arid regions, especially in the desert; the rock-inhabiting species, such as *P. bolanderi*, *P. euploca*, *P. omphaliza*, *P. placodizans*, *P. polycarpa*, *P. sonorensis*, *P. submarginata*, and most subfruticose species, need a dry microenvironment, but also require high levels of precipitation and light [6,7,10,11,12,13,14,26,27,28,29,30,31,32,33,34,36]. The phylogenetic analyses support species groups that correspond to the substrate. We therefore suggest that the substrate should be considered in the species delimitation of *Peltula*.

## Figures and Tables

**Table 1:** Specimens used for DNA extraction and GenBank accession numbers of all samples used in this study. Regarding the voucher information, B.B. refers to B. Büdel, Kaiserslautern, Germany; M.S. to M. Schultz, Hamburg, Germany; K.K. to K. Kalb, Neumarkt, Germany; DUKE refers to Duke University. Newly generated sequences are in bold.

		GenBank Accession Numbers			
Species	Voucher Information	nrSSU	nrLSU	ITS	RPB2
*Peltula africana*	South Africa, 1990, B.B. 14304b	MF766261	MF766384	MF766343	MF804877
*P. anthracina*1	Brazil, 2010, CGMS 385	—	—	MW267988	—
*P. anthracina*2	Brazil, 2010, CGMS 384	—	—	MW267989	—
*P. auriculata*1	Venezuela, 1992, B.B. 24901	—	DQ832330	DQ832329	—
*P. auriculata*2	Venezuela, 1992, B.B. 24902	MF766262	MF766385	MF766344	MF804860
*P. bolanderi*	Mexico, 1993, B.B. 20196e	MF766263	MF766386	MF766345	MF804891
*P. boletiformis*	South Africa, 2003, B.B. 14911a-1	MF766264	MF766387	MF766346	MF804892
*P. brasiliensis*	South Africa, 1983, B.B. 14083a	MF766298	MF766421	MF766380	MF804874
*P. capensis*	South Africa, 1994, B.B. 14382b 2	MF766265	MF766388	MF766347	MF804893
*P. cataractae*	Dem. Rp. Congo, 1947, B.B. 1329	MF766299	MF766422	MF766381	MF804875
*P. clavata*	Australia, 1987, DUKE 164 (18047a)	MF766266	MF766389	MF766348	MF804861
*P. confusa*	20191485 (HMAS-L 154714)	—	**OP429688**	**OP429718**	—
*P. confusa*	20191619 (HMAS-L 154710)	**OP429768**	**OP429710**	**OP429719**	—
*P. confusa*	20191620 (HMAS-L 154707)	**OP429760**	**OP429685**	**OP429720**	**OP348234**
*P. confusa*	20191621 (HMAS-L 154711)	**OP429761**	**OP429689**	**OP429721**	**OP348228**
*P. confusa*	20191622 (HMAS-L 154708)	**OP429762**	**OP429686**	**OP429722**	**OP348233**
*P. confusa*	20191623 (HMAS-L 154709)	**OP429763**	**OP429690**	**OP429723**	**OP348229**
*P. confusa*	20191646-2 (HMAS-L 154715)	—	**OP429691**	**OP429724**	—
*P. confusa*	20191648 (HMAS-L 154713)	—	**OP429692**	**OP429726**	**OP348231**
*P. confusa*	20191928 (HMAS-L 154716)	**OP429765**	**OP429693**	**OP429727**	**OP348232**
*P. confusa*	20191944 (HMAS-L 154717)	**OP429764**	**OP429687**	**OP429725**	**OP348230**
*P. confusa*	20192000 (HMAS-L 154718)	—	**OP429694**	**OP429728**	**OP348226**
*P. confusa*	20192011 (HMAS-L 154719)	—	**OP429695**	**OP429729**	**OP348227**
*P. confusa*	20192022 (HMAS-L 154750)	—	**OP429696**	**OP429730**	—
*P. congregata*	South Africa, 2003, B.B. 14909b-1	MF766267	MF766390	MF766349	MF804896
*P. coriacea*	South Africa, 2003, B.B. 14500a-1	MF766268	MF766391	MF766350	MF804878
*P. corticola*	Yemen, 2002, M.S. 14201	MF766296	MF766419	MF766378	MF804873
*P. crispatula*	Morocco, 1987, B.B. 21001a	MF766269	MF766392	MF766351	MF804862
*P. cylindrica*	South Africa, 2003, B.B. 14920a-1	MF766270	MF766393	MF766352	—
*P. euploca*	Mexico, 1993, B.B. 20162a	MF766271	MF766394	MF766353	MF804879
*P. euploca* ssp. *sorediosa*	South Africa, 2003, B.B. 14921c-1	MF766272	MF766395	MF766354	MF804880
*P. farinosa*	Mexico, 1993, B.B. 20119a	MF766273	MF766396	MF766355	MF804898
*P. hassei*	South Africa, 1994, B.B. 14354a	MF766283	MF766406	MF766365	MF804895
*P. imbricata*	Australia, 1987, B.B. 18060a	MF766274	MF766397	MF766356	MF804899
*P. impressa*	Mexico, 1993, B.B. 20140f	MF766275	MF766398	MF766357	MF804863
*P. impressula*	China, 2019, HMAS-L 154740	**OP429747**	**OP429698**	**OP429737**	—
*P. impressula*	China, 2019, HMAS-L 154741	**OP429746**	**OP429697**	**OP429738**	—
*P. impressula*	China, 2019, HMAS-L 154742	—	**OP429699**	**OP429743**	—
*P. impressula*	China, 2017, HMAS-L 154743	**OP429748**	**OP429700**	**OP429739**	—
*P. impressula*	China, 2017, HMAS-L 154744	**OP429749**	**OP429701**	**OP429740**	—
*P. impressula*	China, 2017, HMAS-L 154745	**OP429750**	**OP429702**	**OP429741**	—
*P. impressula*	China, 2017, HMAS-L 154746	**OP429751**	**OP429703**	**OP429742**	—
*P. inversa*	Namibia, 2001, Pretoria 15058	MF766276	MF766399	MF766358	MF804881
*P. leptophylla*	Mexico, 1993, B.B. 20128a	MF766277	MF766400	MF766359	MF804864
*P. lingulata*	South Africa, 1994, B.B. 14452a	MF766278	MF766401	MF766360	MF804882
*P. lobulata*	China, 2019, HMAS-L 145468	—	MT499313	MT499291	—
*P. lobulata*	China, 2019, HMAS-L 145469	—	MT499314	MT499292	—
*P. lobulata*	China, 2019, HMAS-L 145470	—	MT499315	MT499293	—
*P. marginata*	South Africa, 2003, B.B. 14920d-1	MF766279	MF766402	MF766361	MF804883
*P. michoacanensis*	Mexico, 1993, B.B. 20140l	MF766280	MF766403	MF766362	MF804900
*P. obscuratula*	20191465 (HMAS-L 154747)	**OP429752**	**OP429708**	**OP429735**	—
*P. obscuratula*	20191491 (HMAS-L 154748)	—	**OP429707**	**OP429734**	—
*P. obscuratula*	20191493 (HMAS-L 154749)	**OP429753**	**OP429709**	**OP429736**	—
*P. obscuratula*	Morocco, 1987, B.B. ex Murcia	MF766284	MF766407	MF766366	MF804867
*P. omphaliza*	Mexico, 1993, B.B. 20148b	MF766285	MF766408	MF766367	MF804884
*P. patellata*	Mexico, 2003, M.S. 16254b	MF766286	MF766409	MF766368	MF804868
*P. placodizans*	Mexico, 1993, B.B. 20112a	MF766287	MF766410	MF766369	MF804885
*P. polycarpa*	China, 2019, HMAS-L 145471	MT499282	MT499319	MT499300	**OP348224**
*P. polycarpa*	China, 2019, HMAS-L 145472	MT499286	MT499320	MT499301	—
*P. polycarpa*	China, 2019, HMAS-L 145473	MT499287	MT499321	MT499302	**OP348225**
*P. polyphylla*	China, 2019, HMAS-L 145475	—	MT499326	MT499303	—
*P. polyphylla*	China, 2019, HMAS-L 145474	—	MT499325	MT499304	**OP348221**
*P. polyspora*	20190012 (HMAS-L 154721)	**OP429758**	**OP429706**	**OP429732**	—
*P. polyspora*	20190196 (HMAS-L 154723)	**OP429757**	**OP429704**	**OP429733**	—
*P. polyspora*	20191252 (HMAS-L 154724)	**OP429759**	**OP429705**	**OP429731**	—
*P. psammophila*	Canary Islands, 1985, BM 761074	MF766288	MF766411	MF766370	MF804869
*P. pseudoboletiformis*	China, 2019, HMAS-L 145476	MT499288	MT499322	MT499297	**OP348222**
*P. pseudoboletiformis*	China, 2019, HMAS-L 145478	MT499289	MT499323	MT499298	—
*P. pseudoboletiformis*	China, 2019, HMAS-L 145477	MT499290	MT499324	MT499299	—
*P. radicata*	Yemen, 2002, M.S. 14241a	MF766289	MF766412	MF766371	MF804870
*P. richardsii*	Mexico, 1993, B.B. 20194a	MF766290	MF766413	MF766372	MF804871
*P. rodriguesii*	Namibia, 1990, B.B. 15901	MF766291	MF766414	MF766373	MF804872
*P. santessonii*	South Africa, 2003, B.B. 14912b-1	MF766292	MF766415	MF766374	MF804886
*P. sonorensis*	Mexico, 1993, B.B. 20196d	MF766293	MF766416	MF766375	MF804887
*P. steppae*	Venezuela, 1989, K.K. 23948	MF766297	MF766420	MF766379	MF804890
*P. submarginata*	China, 2019, HMAS-L 145480	MT499283	MT499316	MT499294	—
*P. submarginata*	China, 2019, HMAS-L 145479	MT499284	MT499317	MT499296	**OP348223**
*P. submarginata*	China, 2019, HMAS-L 145481	MT499285	MT499318	MT499295	—
*P. subpatellata*	20190050 (HMAS-L 154729)	—	**OP429679**	**OP429712**	—
*P. subpatellata*	20190053 (HMAS-L 154730)	—	**OP429680**	**OP429713**	—
*P. subpatellata*	20190112 (HMAS-L 154732)	**OP429754**	**OP429681**	**OP429714**	—
*P. subpatellata*	20190143 (HMAS-L 154733)	—	**OP429682**	**OP429715**	—
*P. subpatellata*	XL2017268 (HMAS-L 154738)	**OP429755**	**OP429683**	**OP429716**	—
*P. subpatellata*	XL2017279 (HMAS-L 154737)	**OP429756**	**OP429684**	**OP429717**	—
*P. tortuosa*	Venezuela, 1996, B.B. 24039b	MF766294	MF766417	MF766376	—
*P. umbilicata*	South Africa, 2003, B.B. 14901a-1	DQ782887	DQ832334	DQ832333	DQ832335
*P. zahlbruckneri*	Mexico, 1993, B.B. 20157a	—	MF766418	—	MF804889
*Peccania terricola*	China, Xinjiang University, 201899118	—	OM523033	OM523029	—
*Peccania sp*	China, 2019, HMAS-L 154764	**OP429766**	**OP429711**	**OP429744**	—
*Peccania sp*	China, 2019, HMAS-L 154765	**OP429767**	—	**OP429745**	—

## Data Availability

All data used in this study are reported in the paper.

## Supplementary Materials

The following supporting information can be downloaded at: https://www.mdpi.com/article/10.3390/biology11101518/s1, Figure S1: The maximum likelihood tree of *Peltula* species based on the nrSSU sequences; Figure S2: The maximum likelihood tree of *Peltula* species based on the ITS sequences; Figure S3: The maximum likelihood tree of *Peltula* species based on the nrLSU sequences; Figure S4: The maximum likelihood tree of *Peltula* species based on the RPB2 sequences. Figure S5: The Bayesian tree of *Peltula* species based on the concatenated ITS + nrSSU + nrLSU + RPB2 data set.
