# Supplementary material for: Two New Species and Two New Records of the Lichen-Forming Fungal Genus Peltula (Ascomycota: Peltulaceae) from China
Source: Biology (Basel). 2022 Oct 17;11(10):1518. doi: 10.3390/biology11101518 (PMC9598497; doi:10.3390/biology11101518)
Supplement: Supplementary file 1 [file biology-11-01518-s001.zip › biology-1919239-supplementary.pdf]

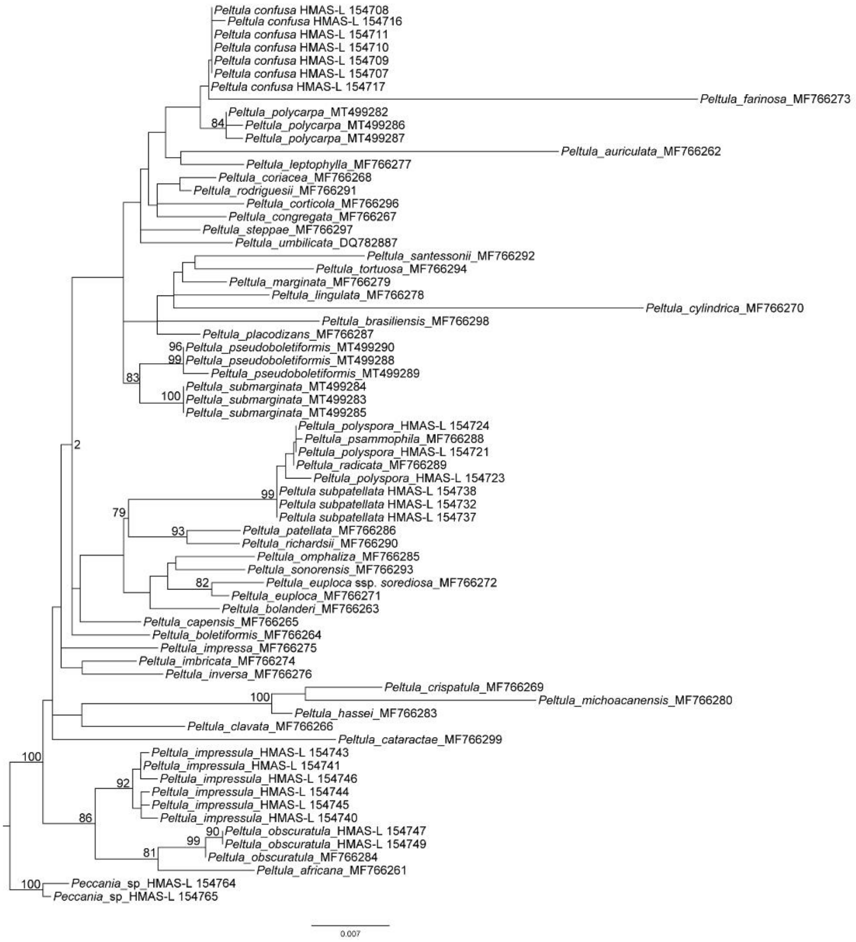

Figure S1. The maximum likelihood tree of *Peltula* species based on the nrSSU sequences. The numbers in each node represent bootstrap support (BS). Bootstrap values ≥ 75 were plotted on the branches of the RAxML tree. The clades corresponding to the new species and the new records are in bold. Scale = 0.007 substitution per site.

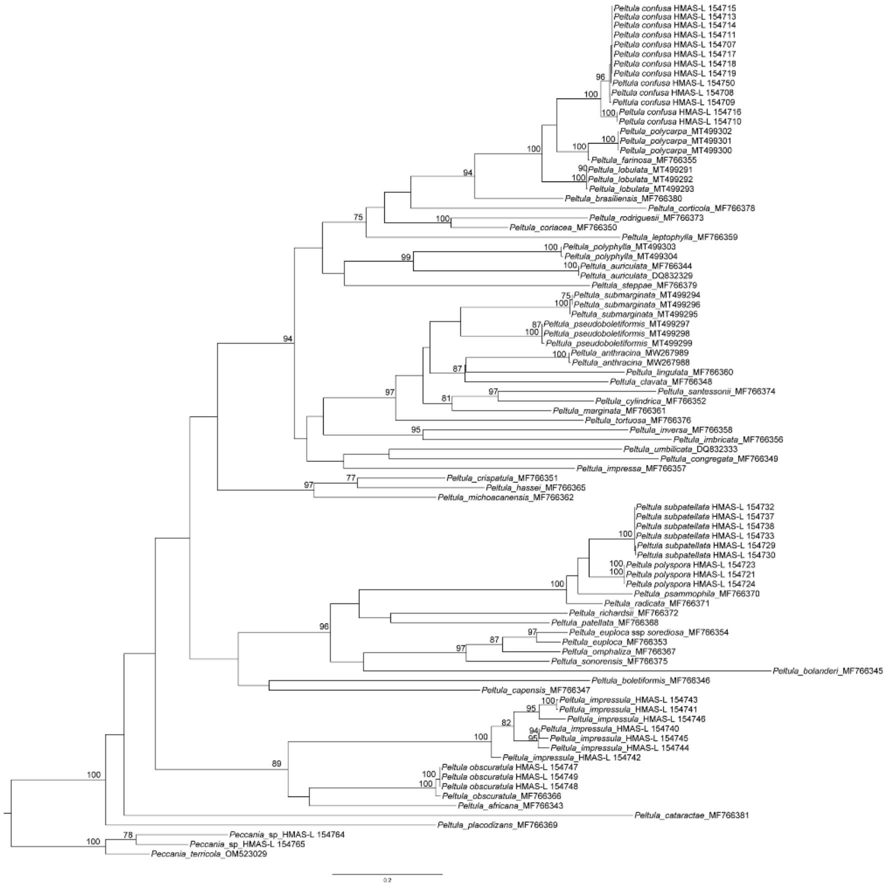

Figure S2. The maximum likelihood tree of *Peltula* species based on the ITS sequences. The numbers in each node represent bootstrap support (BS). Bootstrap values ≥ 75 were plotted on the branches of the RAxML tree. The clades corresponding to the new species and the new records are in bold. Scale = 0.2 substitution per site.

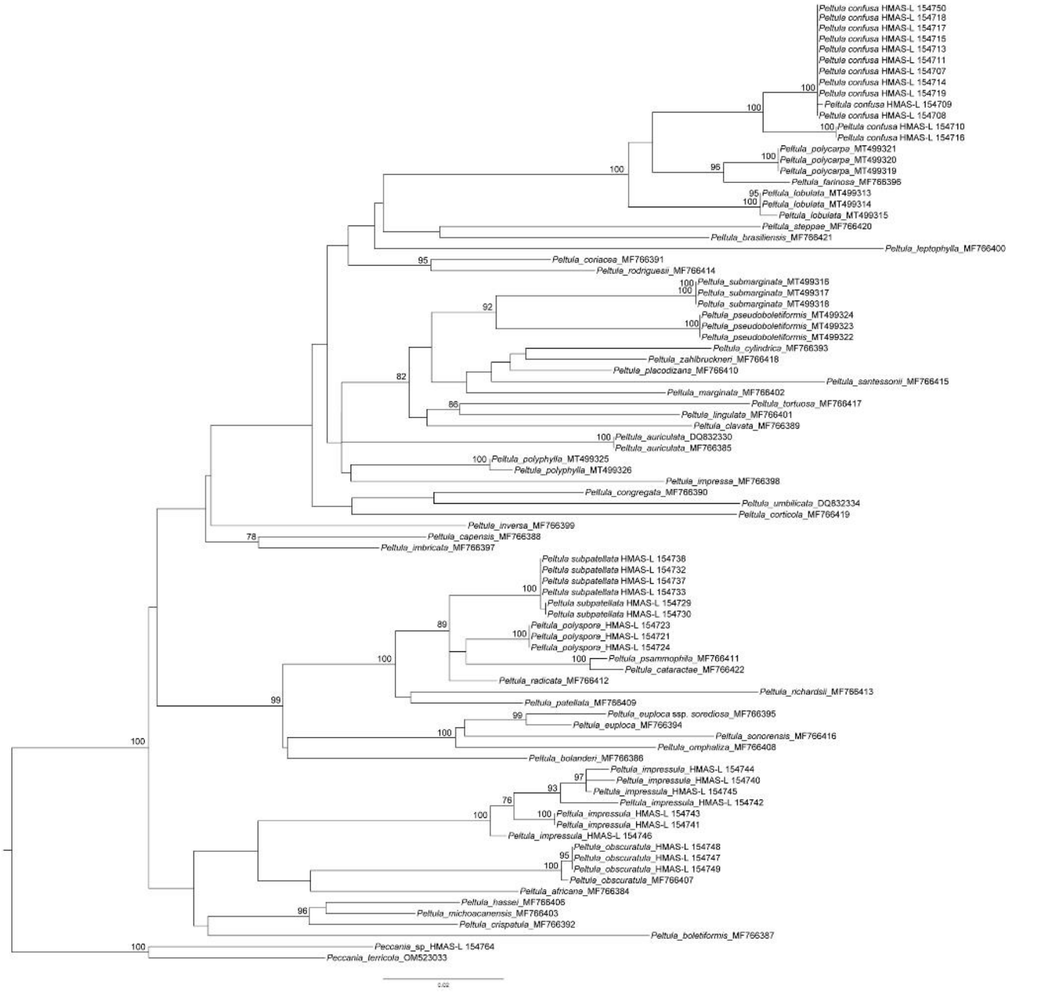

Figure S3. The maximum likelihood tree of *Peltula* species based on the nrLSU sequences. The numbers in each node represent bootstrap support (BS). Bootstrap values ≥ 75 were plotted on the branches of the RAxML tree. The clades corresponding to the new species and the new records are in bold. Scale = 0.02 substitution per site.

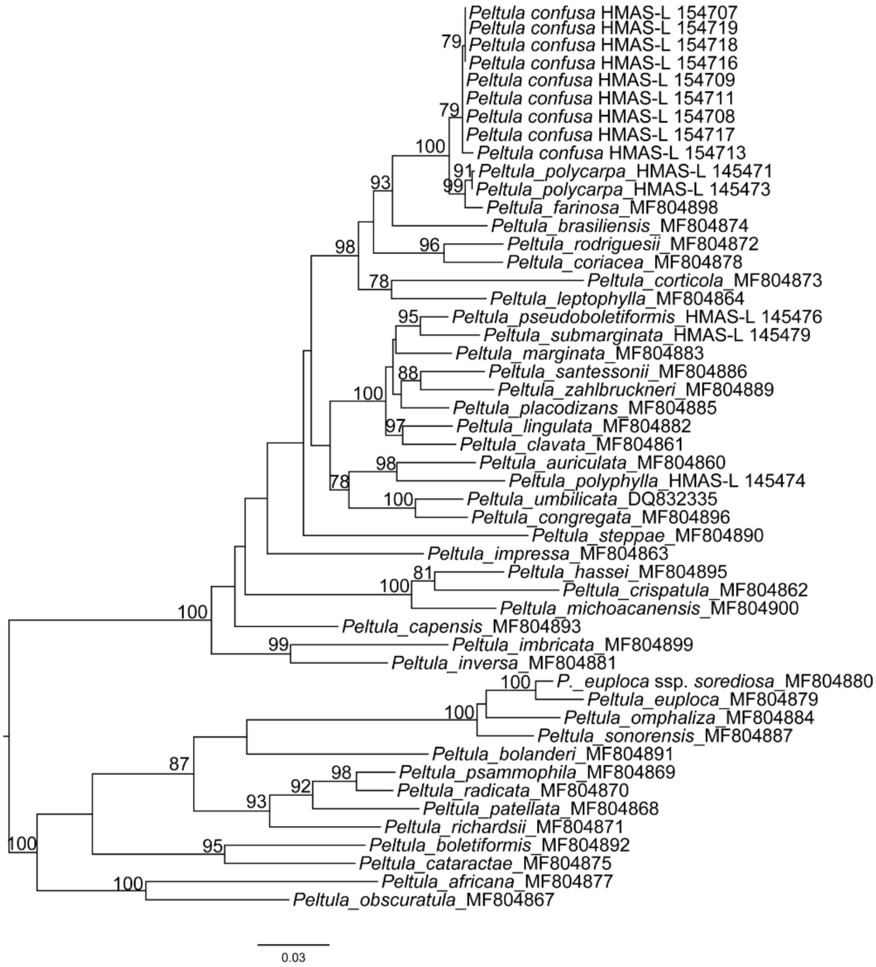

Figure S4. The maximum likelihood tree of *Peltula* species based on the RPB2 sequences. The numbers in each node represent bootstrap support (BS). Bootstrap values ≥ 75 were plotted on the branches of the RAxML tree. The clades corresponding to the new species and the new records are in bold. Scale = 0.03 substitution per site.

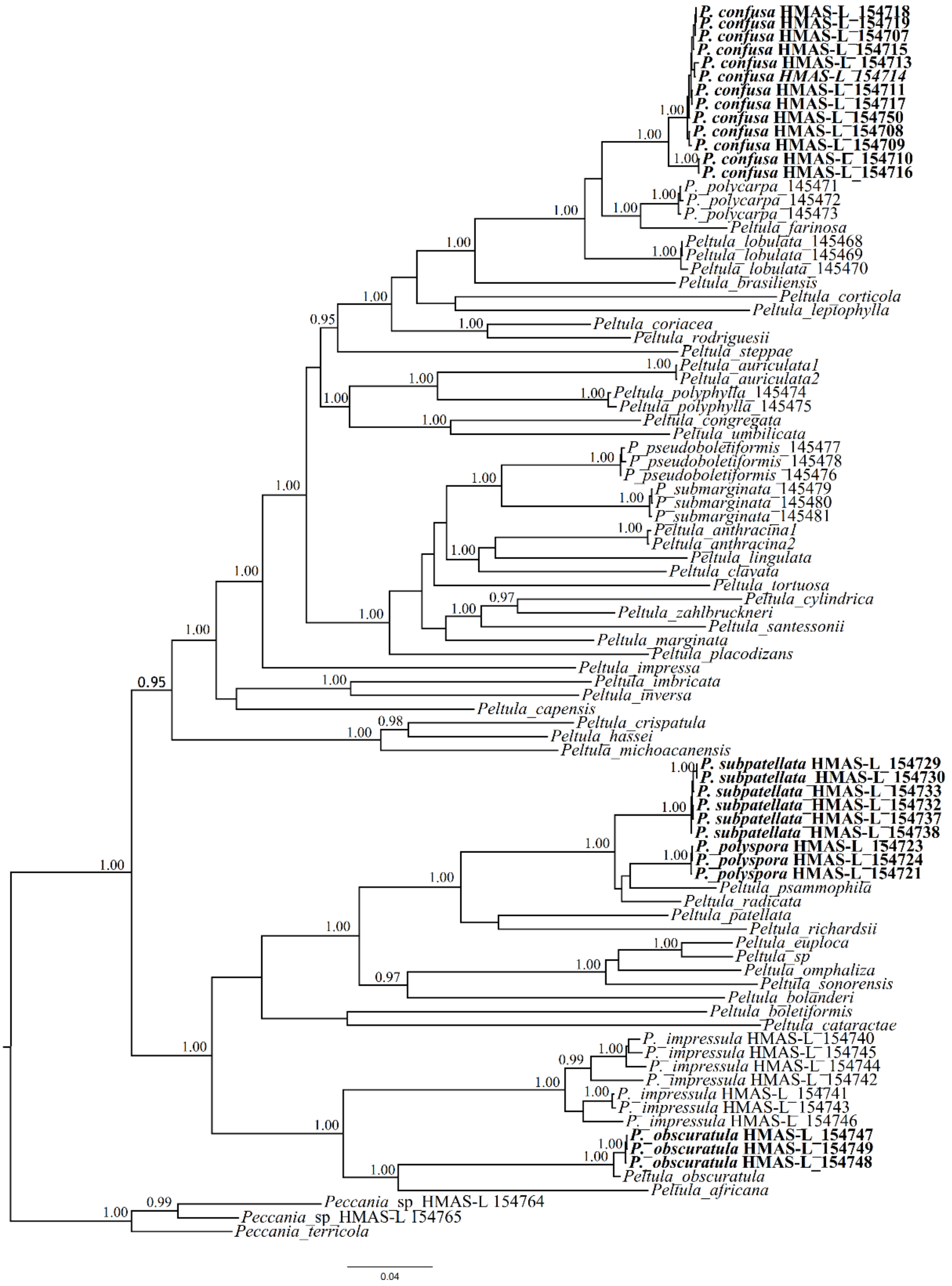

Figure S5. The Bayesian tree of *Peltula* species based on the concatenated ITS + nrSSU + nrLSU + RPB2 data set. The numbers in each node represent posterior probability (PP) values. Posterior probability values ≥ 0.95 were plotted on the branches of the MrBayes tree. The clades corresponding to the new species and the new records are in bold. Scale = 0.04 substitution per site.
